# ZAP-70 Shapes the Immune Microenvironment in B Cell Malignancies

**DOI:** 10.3389/fonc.2020.595832

**Published:** 2020-10-27

**Authors:** Jingyu Chen, Andrew Moore, Ingo Ringshausen

**Affiliations:** Department of Haematology, Jeffrey Cheah Biomedical Centre, Wellcome Trust/MRC Cambridge Stem Cell Institute, University of Cambridge, Cambridge, United Kingdom

**Keywords:** ZAP-70, tumor microenvironment, immunotherapy, B cell lymphoma, CLL

## Abstract

Zeta-chain-associated protein kinase-70 (ZAP-70) is a tyrosine kinase mainly expressed in T cells, NK cells and a subset of B cells. Primarily it functions in T cell receptor (TCR) activation through its tyrosine kinase activity. Aberrant expression of ZAP-70 has been evidenced in different B cell malignancies, with high expression of ZAP-70 in a subset of patients with Chronic Lymphocytic Leukemia (CLL), associating with unfavorable disease outcomes. Previous studies to understand the mechanisms underlying this correlation have been focused on tumor intrinsic mechanisms, including the activation of B cell receptor (BCR) signaling. Recent evidence also suggests that ZAP-70, intrinsically expressed in tumor cells, can modulate the cross-talk between malignant B cells and the immune environment, implying a more complex role of ZAP-70 in the pathogenesis of B cell malignancies. Meanwhile, the indispensible roles of ZAP-70 in T cell and NK cell activation also demonstrate that the autologous expression of ZAP-70 in the immune environment can be a central target in modulation of tumor immunity. Here we review the evidences of the link between ZAP-70 and tumor immunology in the microenvironment in B cell malignancies. Considering an emerging role of immunotherapies in treating these conditions, understanding the distinct molecular functions of ZAP-70 in a broader cellular context could ultimately benefit patient care.

## Introduction

Zeta-chain-associated protein kinase-70 (ZAP-70) is a tyrosine kinase mainly expressed in T cells and NK cells (1, 2). The function of ZAP-70 in T cell receptor (TCR) activation through its tyrosine kinase activity has been well-studied through pioneering works by the Weiss laboratory and others [for review see (3)]. In the early 2000s, the aberrant high expression of ZAP-70 was identified in a subset of Chronic Lymphocytic Leukemia (CLL) patients (4), which turned out to also reflect an unfavorable clinical outcome (5). Much work has been done to establish ZAP-70 as a prognostic marker in CLL, assuming that assessment of its expression was somehow less time and labor-consuming than *IGHV* mutation analyses (6). However, the variation of expression levels and the lack of harmonized tests have hampered this development (7), consequently ZAP-70 expression is not routinely assessed to guide clinical decisions. Subsequent studies further revealed the expression of ZAP-70 in other B cell malignancies, such as Acute Lymphoblastic Leukemia (ALL), Burkitt-lymphoma and Mantle Cell Lymphoma (MCL) (8, 9). Although studies have shown the involvement of ZAP-70 in IgM-mediated B cell receptor (BCR) signaling in CLL, the role of ZAP-70 in the pathogenesis of CLL and other B cell malignancies is still arguable. Recently studies have implied that tumor intrinsic ZAP-70 expression modulates the cross-talk between malignant B cells and their environment, suggesting a new angle to understand the role of ZAP-70 in these diseases. We will review here how ZAP-70 expression in malignant B cells has an impact on cell migration, innate immune response, and T cell infiltration. In contrast, its expression in T cells and NK cells can affect tumor immune responses. Therefore, targeting ZAP-70 may exert anti-tumor effects not only through the modulation of signaling cascades in malignant B cells, but also through inhibition of cells resident or recruited to the tumor microenvironment.

## ZAP-70 Expression in B Cell Malignancies

The expression of ZAP-70 in B cell malignancies was first detected in CLL with 20–80% of leukemic B cells having ZAP-70 expression levels equivalent to autologous CD3+ T cells in patients, correlating with unmutated *IGHV* gene and poor clinical outcomes (5, 6, 10, 11). Notably, the expression of ZAP-70 in CLL cells frequently varies across the entire clone and a somewhat arbitrary threshold of >20% is required to classify a patient by flow-cytometry as “ZAP-70-positive.” Importantly, the expression levels of ZAP-70 in CLL cells are relatively stable over time (6, 10, 12). The aberrant ZAP-70 expression has further been found to associate with sIgM expression in CLL (13), which further suggested an essential role of ZAP-70 in CLL pathogenesis and progression. Importantly, discordant cases of ZAP-70 expression in *IGHV*- mutated CLL indicated that it possesses a higher predictive value for a poor clinical outcome and therefore strongly suggest that it may actively contribute to the pathogenesis (5, 6). In addition to CLL, ZAP-70 is also expressed in a fraction of B-ALL cases, including most of the childhood pre-B cells ALL (14, 15) and adult ALL cases with different maturation phenotypes (9, 16). Notably, ZAP-70 level in ALL is associated with CD38 expression, but no correlation was observed to specific cytogenetic abnormalities (9, 17). Moreover, ZAP-70 expression was identified in a subset of other B cell malignancies, including, Follicular Lymphoma (FL), Mantle Cell Lymphoma (MCL), Hairy Cell Leukemia (HCL), and Diffuse Large B-cell Lymphoma (DLBCL) by western blotting, flow cytometry (14) and immunohistochemistry assessment (8), and in very rare cases of classic Hodgkin lymphoma (18).

The presence of ZAP-70 in subsets of B cell malignancies also with immature phenotypes may reflect their cellular origin, since ZAP-70 expression is also evidenced in normal B cells, especially developing and differentiating B cells. Using a ZAP-70 deficient mouse model, the protein was found to be expressed in pro-B and pre-B cells and to play a role in the process of pro-B to pre-B cells transition in the bone marrow through engaging in the pre-BCR complex formation (19). Notably, ZAP-70 and SYK were functionally redundant in B cell development, since only mice with both ZAP-70 and SYK deficiency displayed a complete B cell developmental block (19, 20). This finding was further supported by a study analyzing B cell populations from human bone marrow, peripheral blood, and tonsils, which found ZAP-70 expression in pro-B and pre-B cells but not in the majority of normal mature B cells (9). Notably, similar to malignant B cells, ZAP-70 expression in normal B cell populations is also modulated by phosphorylation upon BCR activation (14, 21).

Since ZAP-70 is normally not expressed in mature B cells, its expression in CLL and other mature B cell-derived neoplasms likely points to their different cellular origin (9, 15). Interestingly, point mutations in *ZAP-70*, which can result in the lack of ZAP-70 protein expression in human T cells, were not identified in normal human B cells and ZAP-70 negative malignant B cells (9). Therefore, the down-regulation of ZAP-70 through B cell development may represent a physiological process of B cell maturation.

The aberrant high ZAP-70 expression found in some mature B cell malignancies may be caused by epigenetic modulation and clonal evolution during tumor transformation. In CLL, hypomethylation on CpG sites in the *ZAP-70* gene 5′ regulatory regions have been identified to be associated with high ZAP-70 expression and predictive of a poor disease outcome (22–24). Alternative mechanisms leading to the aberrant expression of ZAP-70 relate to tumor-microenvironment mediated induction of ZAP-70: In B cells derived from peripheral blood, which have consistently low ZAP-70 levels, BCR-activating stimuli (e.g., anti-IgM, sCD40L, IL-4, IL-6, and IL-10) upregulate the expression of ZAP-70 (14). Unmethylated CpG oligodeoxynucleotides, which can trigger an innate immune response through TLR9 activation, promote proliferation in a subset of CLL cells, accompanied by ZAP-70 induction (25, 26).

## Tumor ZAP-70 Expression Modulates the Tumor- and Immune Microenvironment

Efforts have been made to understand the molecular role of tumor-intrinsic ZAP-70 expression in B cell malignancies. In CLL, ZAP-70 expression is associated with enhanced BCR signaling upon IgM activation, evidenced by a positive correlation between ZAP-70 expression, phosphorylation of SYK, BLNK, and PLCγ2 and calcium response (4, 27). Notably, the kinase activity of ZAP-70 is dispensable for BCR signaling in CLL, since the phosphorylation of ZAP-70 catalytic sites appears negligible compared to that of SYK (28). In addition an introduced mutation abrogating kinase activity of the ZAP-70 catalytic site had no significant effect on IgM-mediated BCR signaling activation (29). This suggests that the role of ZAP-70 in B cell malignancies is different from that in T cells. Interestingly, despite the dispensable nature of its kinase activity, ectopic expression of ZAP-70 in the Burkitt lymphoma line BJAB enhanced the phosphorylation and activation of BCR-related signaling cascades under conditions of IgM activation (28). These findings have led to the suggestion that ZAP-70 acts mainly as an adaptor protein to recruit downstream protein kinases, such as PI3K, c-Cbl, Cbl-b, and Shc (28). In contrast, in B-ALL, ZAP-70 is constitutively phosphorylated, suggesting the tyrosine kinase activity is continuously involved in ALL biology (16). However, the detailed role of ZAP-70 in B-ALL is still unknown.

**Figure 1:**
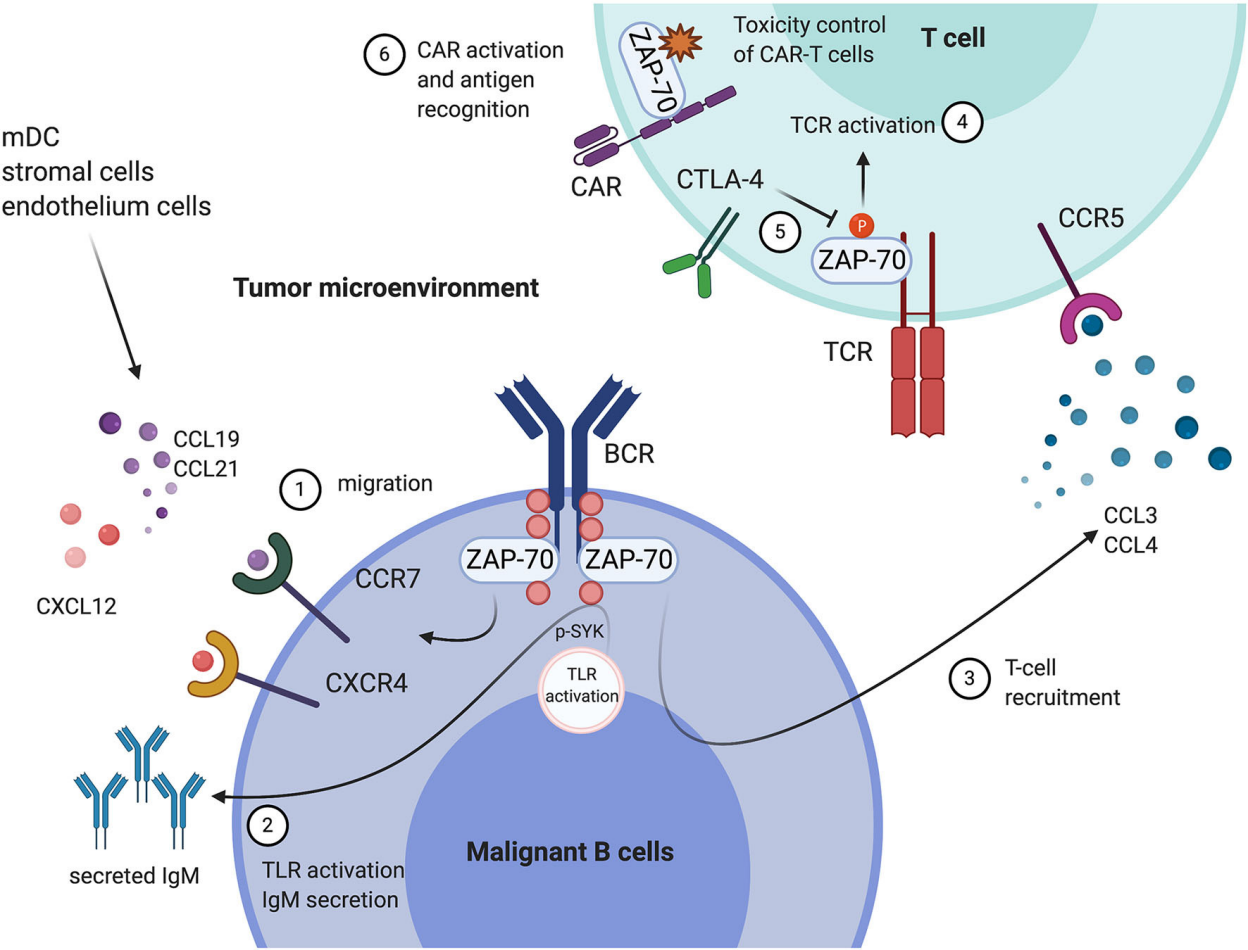
Tumor expression of ZAP-70 modulates the immune microenvironment, and the functional roles of ZAP-70 in environmental T cells. In Chronic Lymphocytic Leukemia (CLL) cells, ZAP-70 mediates BCR signal transduction through its kinase activity or as a scaffold protein recruiting other tyrosine kinases. In addition, ZAP-70 modulates the communication between malignant cells and their tumor microenvironment. (1) ZAP-70 is involved in the regulation of CCR7, CXCR4 expression on CLL cells, which promote the migration of tumor cells toward an environment niche, secreting CCL19, CCL21, and CXCL12; (2) ZAP-70 in CLL is engaged in TLR9 activation-mediated anti-apoptotic effects and cell proliferation, likely through mediating SYK activation and IgM secretion; (3) ZAP-70 expression in CLL cells is associated with high levels of CCL3 and CCL4 secretion, which engage in the recruitment of T cells. In T cells in the tumor microenvironment, (4) ZAP-70 expression and phosphorylation is essential for TCR-mediated T cell activation; (5) CTLA-4 is a negative regulator of ZAP-70 phosphorylation, thus suppressing T cell activation; (6) ZAP-70 expression in CAR-T cells is important for cell proliferation and antigen recognition. This Figure has been created with Biorender.com.

In addition to engaging in tumor cell intrinsic signaling, likely improving the cellular fitness of tumor cells, evidence suggest that ZAP-70 expression is also involved in the cross-talk between malignant B cells and their microenvironment (Figure 1).

### Cell Migration

C-C chemokine receptor type 7 (CCR7) and C-X-C chemokine receptor type 4 (CXCR4) expression on B cells is essential for cell migration and homing during B cell development through the binding to their putative chemokine ligands CCL19/CCL21 and CXCL12, respectively (30). High receptor expression on malignant B cells correlates with advanced disease stage in CLL (31), and in Diffuse Large B cell Lymphoma (DLBCL), associated with increased bone marrow infiltration and poor outcomes (32). Other studies have shown ZAP-70 expression correlates with enhanced T- and B cells migration and chemotaxis in the microenvironment. In a recent study deciphering the molecular cues which modulate inflammation-dependent oligomerization of the chemokine receptor CCR7 in dendritic- and T cells, ZAP-70 has been identified as an interactor of CCR7 under chemokine stimulation, suggesting a role of ZAP-70 in CCR7 related cell migration and chemotaxis (33). This finding is consistent with previous studies showing ZAP-70 expression in CLL cells correlates with CCR7 expression, induced by IgM-mediated ERK activation, thus enhancing the migratory ability to CCL19 and CCL21 (34, 35). A recent study has further evidenced this in CLL patients, and observed that ZAP-70 positive CLL cells migrated more to CCL19, CCL21, and CXCL12 by controlling the chemokine-driven clustering of the integrins VLA-4 and LFA-1 (36). Moreover, ZAP-70 expression also correlates with CCR7/CXCR4 expression in B cell precursor ALL disease and here promotes migration toward CCL19/CXCL12 in the central nervous system (37).

### Innate Immune Responses

Besides BCR mediated signals, Toll-like receptor (TLR) signaling, which can bridge innate and adaptive immune responses, has been found to play a role in CLL activation and proliferation (38, 39). Interestingly, ZAP-70 appears to play a role to determine the environmental triggered TLR response in CLL: A recent study from our lab has elucidated that expression of ZAP-70 in CLL is strongly predictive of TLR9 agonists-mediated anti-apoptotic effects and cell proliferation, likely through mediating SYK activation, IgM secretion and Bim degradation (26).

### T Cell Infiltration

Mounting evidence indicates that the ZAP-70 expressed in tumor cells has ramifications for the composition of immune cells in the microenvironment, especially for the number of infiltrating T cells. In studies comparing the immune-phenotype of ZAP-70 positive and ZAP-70 negative CLL patients, tumor ZAP-70 expression was associated with increased CD4 central memory T cells and CD3/CD69+ T cells with decreased CD4/CD8 ratio in the peripheral blood (40–42). However, since high ZAP-70 expression is normally observed in only a subpopulation of CLL cells and varies substantially between patients, it is possible that subtle changes in different T cell populations between ZAP-70 positive and negative patients are partly impacted by inconsistencies in the definition of ZAP-70 positive in these studies. Interestingly, studies have evidenced that CLL cells secrete the C-C motif chemokine ligands, CCL3 and CCL4, which enable the recruitment of T cells and monocytes, under the stimulation of IgM and in co-culture with nurse-like cells (NLC) (43). In addition, in CLL, ZAP-70 positive patients have significantly higher CCL3 and CCL4 plasma levels (43), and CCL3 plasma levels correlate with other risk factors (44). These findings suggest a potential role of tumor autologous ZAP-70, mediating immune-responses and fostering a tumor-supportive microenvironment through modulation of the expression of T cell chemokines.

## ZAP-70 Expression in T Cells and NK Cells, and Their Roles in B Cell Malignancies

Immunotherapy, including checkpoint blockade inhibition and cell-based immunotherapies, is a fast developing area in cancer treatment. Such treatment modalities have been applied to treat B cell malignancies and demonstrated significant improved outcomes in smaller subsets of patients, who previously relapsed from chemotherapies and targeted therapies (45). Notably, despite showing some promising effects, the molecular mechanisms that inhibit T cell and NK cell activation in B cell malignancies and block anti-tumor immunity are far from being comprehensively described.

**Figure 2:**
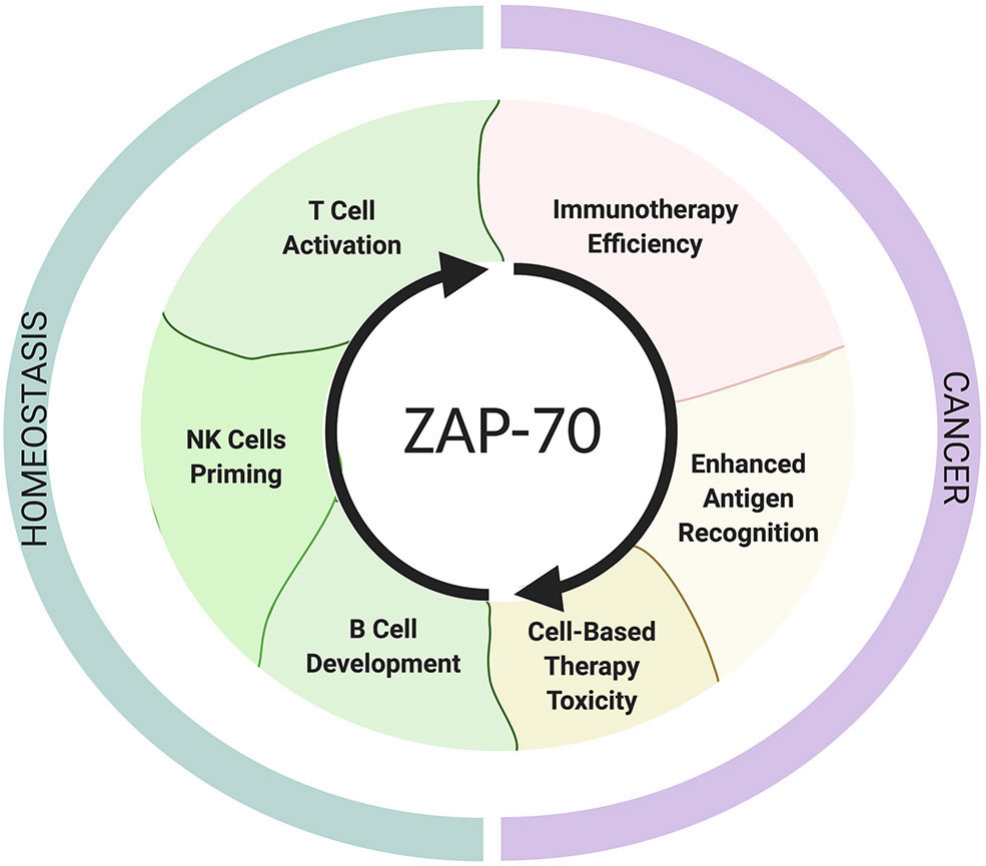
The roles of ZAP-70 in homeostasis and cancer. The schematic shows ZAP-70 involvement in physiological contexts and in cancer. This Figure has been created with Biorender.com.

Because of its indispensible role for TCR activation, deficiency, or aberrantly high expression of ZAP-70 in T cells expectedly result in immune deficiency [ZAP-70-related severe combined immunodeficiency syndromes (SCID)] (46) and autoimmunity, respectively (47). To date, it remains an important but unanswered question whether ZAP-70 expression levels in T and NK cells are associated with patient responses to immunotherapies, but an increasing amount of evidence suggests that ZAP-70 deficiency or inhibition can contribute to impaired tumor-surveillance (Figure 2).

### T Cells

Structurally, ZAP-70 has been found to play a central role in immunological synapse formation in cytotoxic T lymphocytes (CTL) (48). CTLA-4 is a well-established inhibitory checkpoint for T cell activation (49). It has been suggested that the inhibition of ZAP-70 tyrosine-phosphorylation is a mechanism of CTLA-4 mediated suppression of CD4+ T activation, indicating a central role of ZAP-70 kinase activity in T cell activation and anti-tumor immune responses (50). In a recent study the GTPase-activating protein (GAP) Rasal1, which inhibits ZAP-70, has been identified to suppress anti-tumor immune-responses. Antagonizing ZAP-70 inhibition by siRNA against Rasal1 increased the number of CD8+ tumor-infiltrating T-cells expressing granzyme B and interferon gamma (no ‘1') and enhanced tumor killing (51). Notably, in a DLBCL case, ZAP-70 deficiency caused complete ablation of the CD8 population in the tumor environment (52), suggesting a profound effect of ZAP-70 in tumor immune-responses.

### NK Cells

Recently, NK cells have been applied for cancer-immunotherapies, benefitting from its antigen-independent host immune responses and cytotoxicity against malignant cells (53). ZAP-70 is a kinase that is also involved in the activation of NK cells upon engaging with ligands on targeted cells (54) and downregulation of ZAP-70 is associated with inhibition of NK cell responses under prolonged activation and continuous DNA damage stress (55). A very recent study revealed that ZAP-70 is engaged in immunomodulatory drug pomalidomide induced granzyme-B secretion and cytolytic activity of NK cells (56). However, ZAP-70-independent pathways exist which modulate NK cell mediated cytotoxicity, primarily through signaling modulated by non-ITAM-based receptors, like NKG2D (57). It has also been described that NK cells from SYK^−/−^ ZAP-70^−/−^ mice still maintain natural cytotoxicity, which suggests a redundant role of ZAP-70 in this process, despite driving the activation of NK cell receptor signaling (58).

### CAR-T Cells

Chimeric antigen receptor T cells (CAR-T) are T cells expressing artificial T cell receptors which contain both tumor specific- as well as T cell activating motifs (59). Promising results from clinical trials had led to several CAR-T cell therapies approved by the United States Food and Drug Administration and European Medicines Agency for treating relapsed or refractory B cell malignancies (60). In spite of the similarity between chimeric antigen receptors (CARs) and natural TCRs, reduced efficiency of antigen-recognition and affinity remain major issues in CAR-T cell therapies. Third generation CAR-T cells are potentially more efficient than second generation through engaging additional co-stimulatory molecules. Evidence from a comparative study indicates that activation and phosphorylation of ZAP-70 in CAR-T cells is associated with enhanced cell proliferation and expansion of third generation CARs, containing both CD28 and 4-1BB motifs, compared to second generation CARs (61). The importance of ZAP-70 in CAR-T activation has been further addressed by a very recent study: Using quantitative single-molecule live-cell imaging, CAR-T cells have been shown to have ~1,000 times reduced antigen sensitivity compared to normal T cells, and data suggest that the underlying mechanism relates to reduced recruitment of ZAP-70 to CARs. This study enlightens the importance of ZAP-70 in CAR-T activation and suggests it as a promising target for improving CAR-T antigen recognition (62).

## ZAP-70 as Therapeutic Target

Considering the importance of ZAP-70 in T cell and NK cell activation, great effort has been put to target ZAP-70 in order to control diseases derived from abnormal T or NK cell activation, such as immune disorders and autoimmune diseases (3, 63). ZAP-70 has been found not only to function through its kinase activity, but also as an important scaffold protein to associate with TCR or BCR related molecules, independent of its catalytic activity (28, 64), suggesting that kinase-inhibition may not completely abolish protein function. Several *in vitro* studies have previously investigated inhibitors which can suppress ZAP-70 kinase activity or disrupt its protein-binding ability to access downstream TCR related activators (65, 66). These inhibitors have been well-described in a recent review (63).

Although the expression of ZAP-70 in tumor cells has been linked to a dismal outcome, there have only been few attempts to inhibit ZAP-70 as a treatment, partly because the biological functions of ZAP-70 in B cell malignancies remain elusive. Tyrosine kinase inhibitors have been assessed to treat ZAP-70 positive CLL, for example, gefitinib has been tested for inducing apoptosis of ZAP-70 positive CLL cells and cell lines *in vitro*. These studies demonstrated that gefitinib inhibits the basal and BCR activation-mediated phosphorylation of ZAP-70 at the micromolar level and that ZAP-70 expression sensitizes cells to gefitinib induced cell apoptosis (67). However, it is arguable whether these pro-apoptotic effects of gefitinib were achieved through the inhibition of ZAP-70 or other related tyrosine kinases, such as SYK.

While ZAP-70 constitutes an interesting and attractive target for therapeutic interventions in cancer patients, especially in those with aberrant expression in B cell malignancies, the simultaneous inhibition of T and NK cells appears to be inevitable and may be less desirable and potentially even harmful. While T cell subsets may promote tumorigenesis (e.g., through CD40 stimulation) and their inhibition may therefore be therapeutically beneficial, blockage of cytotoxic T cells and NK cells may be less so. Whether different immune cells display different susceptibilities to ZAP-70 inhibition, thus allowing for a wide-enough therapeutic window of antagonists to be beneficial, is unknown, but at least seems possible. We believe this is a substantial problem to be considered in the design of ZAP-70 directed therapies.

Immunotherapies, including CAR-T cells, immune checkpoint blockade, and adaptive T cell therapies, have been applied in clinical treatment for B cell malignancies. The safe and precise control of over-reactions of anti-tumor immune responses has been a major issue for the toxicity of cell-based immunotherapies (68). Based on the essential role of ZAP-70 in TCR activation, some studies suggest targeting ZAP-70 in order to control effector T cells, which could potentially be applied for developing safer adaptive T cell therapies (Figure 1). A recent study defined ZAP-70 as a target to control the toxicity caused by over-reacting CAR-T cells such as cytokine release syndrome (CRS). Dasatinib, a tyrosine kinase inhibitor has been found to attenuate CAR-T toxicity by suppressing ZAP-70 activation (69). However, inhibition of other kinases by dasatinib, such as Abl and Src tyrosine kinases, may likely contribute. An engineered ZAP-70 construct has been established by the Weiss lab to specifically study the role and requirement of ZAP-70 kinase activity in different biological processes. The so-called analog-sensitive ZAP-70 mutant (ZAP-70 AS), which contains an engineered binding pocket around the kinase domain, sensitive to an analog of the small molecule kinase inhibitor PP1, conserves the normal ZAP-70 catalytic activity and can be specifically inhibited (70). This specificity has a great potential to be applied for the safe control of adaptive cell-based immunotherapies (71).

## Conclusion

ZAP-70 is not only critical for T cell and NK cell activation, but also associated with poor outcomes of B cell malignancies, especially in CLL. Tumor intrinsic expression of ZAP-70 in B cell malignancies has been shown to enhance cellular signals under ligand stimulated BCR activation. However, the underlying mechanisms known so far cannot fully explain the correlation between ZAP-70 expression and dismal outcome. More evidence has pointed to ZAP-70 driven environmental changes, which may play a central role for triggering innate immune responses and immune cell infiltration (Figure 1 and Table 1).

**Table 1:** Cell-type specific functions of ZAP-70 in B cell malignancies.

	**Cell types**	**Proposed functions**
Malignant B cells	CLL	✧ ZAP-70 enhances BCR signaling upon IgM activation; ✧ Interactions with BCR-related proteins; ✧ ZAP-70 correlates with CCR7, CXCR4 expression and enhanced cell migration; ✧ Modulation of TLR-induced response through mediating SYK activation, IgM secretion, and Bim degradation; ✧ Associates with CCL3 and CCL4 secretion and T cell infiltration in the tumor microenvironment.
	ALL	✧ Constitutively phosphorylated, detailed role of ZAP-70 in ALL is unknown.
	Others	✧ Undefined
Tumor-environment immune cells	T cells	✧ Essential for TCR activation through its tyrosine kinase activity; ✧ Plays central role in immunological synapse formation in CTL. ✧ CTLA-4 in CD4+ T cells inhibits ZAP-70 activity.
	NK cells	✧ NK cell activation upon receptor engagement; ✧ May be redundant in NK cell mediated cytotoxicity.
Immunotherapy	CAR-T cells	✧ Associates with enhanced cell proliferation and expansion in the 3rd generation CAR-T cells contacting both CD28 and 4-1BB; ✧ Reduced recruitment of ZAP-70 to CARs is associated with less antigen sensitivity compared to normal T cells.

Growing evidence also indicates that the modulation of ZAP-70 activity can be applied to control T cell activation, which has translational potential to mitigate the toxicity associated with cell-based immunotherapies (Figure 1). However, since most of the evidence has only been compiled from *in vitro* experiments, more *in vivo* studies are needed to fully characterize whether such therapies can be applied in a clinical setting.

A thorough review of the published evidences focusing on defining the role of ZAP-70 in health and disease clearly indicates that it remains an attractive target for therapeutic interventions, more than ever. More experimental evidence is needed to fully understand the biology behind ZAP-70 in B cell malignances in a holistic cellular approach. The simultaneous targeting of ZAP-70 in tumor cells, T and NK cells, may be beneficial in some instances, but also bears the risk to promote tumor growth through impairing immune surveillance.

## Author Contributions

JC and IR designed and wrote this review. AM provided critical editing on the manuscript and the graphs. All authors contributed to the article and approved the submitted version.

## Conflict of Interest

The authors declare that the research was conducted in the absence of any commercial or financial relationships that could be construed as a potential conflict of interest.
